# Scientific Items

**Published:** 1884-11-20

**Authors:** 


### SCIENTIFIC ITEMS.

**The Oldest Living Microcephalic.**—At a recent sitting of the Berlin Medical Society, Professor Virchow exhibited a girl, aged fourteen, with a slight though normally developed figure, but with a diminutive head, scarcely as large as a man's fist. She came from Offenbach, and was introduced by her mother, a tall, large-boned woman. Her face is not larger than that of a new-born child, with a sharply projecting nose and prominent jaws; her complexion is delicate, and her features resemble those of a bird of prey. The size of the brain in this diminutive skull is proportionately small, and the intellectual powers are not developed beyond those of a six-months' child. The only word, besides some inarticulate sounds, that the girl can pronounce is mamma. The parents had had seven children, of whom four were microcephalic, but only the one exhibited had lived. When she was at home she sat quietly, and preferred avoiding the society of other children, generally withdrawing into a corner of the room. She ate and drank purely mechanically. Virchow referred to this case as the oldest one living. At the recent meeting of the French Association for the Advancement of Science, Dr. Magitot reported the case of a microcephalic woman, aged thirty, weight seventy pounds. She showed more intelligence than Virchow's case, and could dress, take care of herself, and speak several words.—*New York Medical Record.*

**A Wonderful Clock.**—A clock that will run four hundred days without winding is on exhibition in a down-town window. It stands under a glass shade about fifteen inches high, and is of very simple construction. The only apparent difference between it and a Connecticut clock of novel design is that, in place of a pendulum, it has a time measure of equal exactitude in the shape of a rotary disk of brass containing springs, which wind and unwind as it turns first in one direction and then the other. The clock is a European invention and attracts considerable attention from passers-by. A salesman, in explaining its mechanism to a reporter, said: "There have been former inventions of clocks to run as long a time as this, but never on any such principle or with any success, as they never proved at all accurate. Until this clock was perfected, no one ever produced a clock of plain construction and general usefulness that would run longer than a week or a fortnight at the farthest. A view of this wonder would have delighted Huygens, the father of clockmakers, who produced the first time-piece in the shape of a clock two hundred and fifty years ago.—*New York Mail and Express.*

**Hygienic Sewer Arrangement.**—In view of the proximity of gas-mains to sewers, the plan has recently been devised of inserting what is termed a hygienic gas-furnace in the manholes. The gas is introduced into a little chamber, where it is mixed with a due proportion of air, and supplies some Bunsen burners; immediately above the gas are some fire-clay plates, which soon become heated, while above them are iron divisions. The heat naturally draws the air up from the sewer below; it thus passes through the Bunsen burners, backward and forward over the fire-clay plates and iron divisions, until, at last, it finds its exit in the ventilation chamber or manhole, and hence through the grate into the street. The furnace not only causes a strong current of air from the sewer, but, as it is capable of being heated at from 600° to 700° F., it should destroy all the germ-life that travels with the sewer-gas.—*Pop. Science News.*

**Cholera Germs under the Microscope.**—Some specimens of the peculiar germ which the now famous Dr. Koch has discovered in India, Egypt, and latterly in France, in the bodies of persons who have died of the cholera, and to the presence of which he attributes that terrible scourge, were exhibited for the first time in public yesterday at the Imperial Theater, by the aid of the gigantic microscope which is now being shown there. The germs, which, it is needless to say, are no longer in an active state, have, it is stated, been specially procured recently from the cholera district in the South of France. As magnified some two million times, and shown upon the great screen occupying the entire proscenium of the theater, they appear nearly of the size of the palm of one's hand. They are quite colorless, and in the shape of coils representing circles and shapes not unlike the figure 8, precisely as described in Dr. Koch's work on the subject. The microscope is the same as that which was used at the Crystal Palace for the entertainment of "Les Invisibles," or the wonders of the world unseen by the naked eye, which is now given twice daily at the Imperial Theater.—*London Daily News*, *July* 12.

**Selenetropism.**—Apropos of Prof. Young's interesting article on lunar heat, we may mention that a recent article in Les Comptes Rendus discusses the effect of lunar light in producing movements in plants similar to those caused by sunlight. Ducharte was led, by the influence which a light of very feeble intensity exercises upon heliotropic movements, to vary the experiments by using moonlight. He sowed seeds of plants which were very sensitive to light, such as *Lens esculenta*, *Ervum lens*, *Vicia sativa.* When the plants were a few centimeters in length he put them in a dark place, where he kept them until the night of the experiment. The stalks became slender, long, and white; the leaves developed slightly, with a light yellowish tinge. On three successive nights, when the sky was exceptionally clear, the plants were placed behind a large window with a southern exposure, so that they received the direct light of the moon from 9 P. M. to 3 A. M. From the very beginning of the exposure the stalks began to bend, so as constantly to present their concavity and the terminal leaf-but to the moon, following it in its course.—*Pop. Science News.*

Friends, aid us by bringing our Journal to others' attention.

